# Proteomic Heterogeneity of the Extracellular Matrix Identifies Histologic Subtype-Specific Fibroblast in Gastric Cancer

**DOI:** 10.1016/j.mcpro.2024.100843

**Published:** 2024-09-19

**Authors:** Hyun Jin Lee, Yoonjin Kwak, Yun Suk Na, Hyejin Kim, Mi Ree Park, Jeong Yeon Jo, Jin Young Kim, Soo-Jeong Cho, Pilnam Kim

**Affiliations:** 1 Department of Bio and Brain Engineering, KAIST, Daejeon, Republic of Korea; 2 Department of Pathology, Seoul National University College of Medicine, Seoul, Republic of Korea; 3 Department of Internal Medicine and Liver Research Institute, Seoul National University College of Medicine, Seoul, Republic of Korea; 4 Digital Omics Research Center, Korea Basic Science Institute, Ochang, Republic of Korea; 5 Critical Diseases Diagnostics Convergence Research Center, Korea Research Institute of Bioscience and Biotechnology, Daejeon, Republic of Korea

**Keywords:** gastric cancer, poorly cohesive carcinoma, proteomics, tumor microenvironment

## Abstract

Gastric cancer (GC) is a highly heterogeneous disease regarding histologic features, genotypes, and molecular phenotypes. Here, we investigate extracellular matrix (ECM)-centric analysis, examining its association with histologic subtypes and patient prognosis in human GC. We performed quantitative proteomic analysis of decellularized GC tissues that characterizes tumorous ECM, highlighting proteomic heterogeneity in ECM components. We identified 20 tumor-enriched proteins including four glycoproteins, serpin family H member 1 (SERPINH1), annexin family (ANXA3/4/5/13), S100A family (S100A6/8/9), MMP14, and other matrisome-associated proteins. In addition, histopathological characteristics of GC reveals differential expression in ECM composition, with the poorly cohesive carcinoma-not otherwise specified (PCC-NOS) subtype being distinctly demarcated from other histologic subtypes. Integrating ECM proteomics with single-cell RNA sequencing, we identified crucial molecular markers in the PCC-NOS-specific stroma. PCC-NOS-enriched matrisome proteins and gene expression signatures of adipogenic cancer-associated fibroblasts (CAF_adi_) are closely linked, both associated with adverse outcomes in GC. Using tumor microarray analysis, we confirmed the CAF_adi_ surface marker, ATP binding cassette subfamily A member 8 (ABCA8), predominantly present in PCC-NOS tumors. Our ECM-focused analysis paves the way for studies to determine their utility as biomarkers for patient stratification, offering valuable insights for linking molecular and histologic features in GC.

## Highlights

• Distinct ECM were revealed between normal and GC, emphasizing the ECM heterogeneity.
• Specific ECM proteins were identified for GC subtype characterization and prognosis.
• Fibroblasts with a malignant GC (PCC-NOS) express the ECM linked to poor prognosis.
• Targeting ECM-associated fibroblasts could offer new therapy tailored to GC subtypes.

## Graphical Abstract

**Figure:**
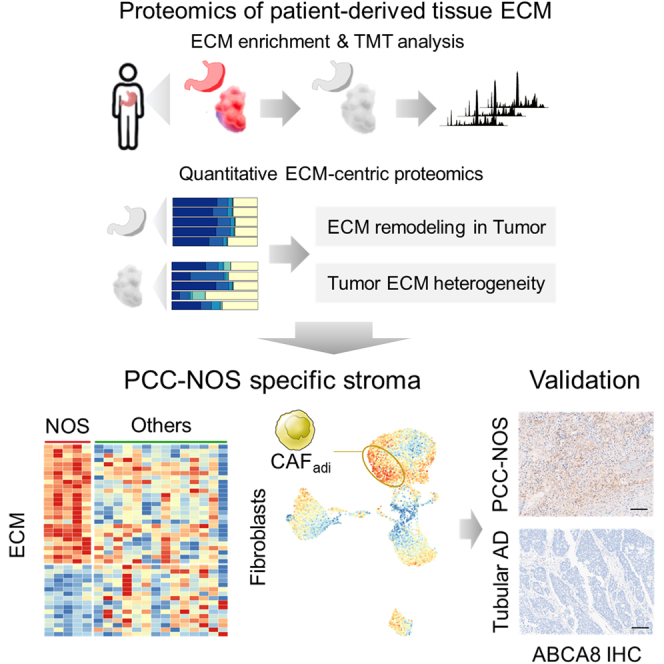

## In Brief

Using proteomic profiling of the extracellular matrix (ECM) of gastric cancer, we have revealed the heterogeneity of the ECM within the tumor tissue and further elucidated the differences in ECM composition based on histologic subtypes, defining enriched ECM profiles specific to certain subtypes. By multimodal analysis, we found a link between the ECM proteins enriched in malignant subtype (PCC-NOS) and a particular type of cell found in tumors, ABCA8+ fibroblast, which is related to worse outcomes in stomach cancer patients.

Gastric cancers (GCs) display significant heterogeneity, leading to multiple efforts to categorize them based on various criteria, including molecular characteristics and histologic features. This multifaceted classification is crucial, as different GC subtypes exhibit variations in genomic and phenotypic profiles. In recent studies, the classification of GC subtypes based on molecular characteristics showed correlations with distinct disease progression patterns and prognosis (1, 2).

In GC, notable contributions have been made by organizations such as the Cancer Genome Atlas (TCGA) project (3) and the Asian Cancer Research Group (2). These groups have distinguished GC patients based on somatic mutations and alterations in the gene expression of neoplastic cells. The TCGA group has classified GC into four subtypes: Epstein-Barr virus-positive, microsatellite unstable, genomically stable, and chromosomal instability. Meanwhile, the Asian Cancer Research Group has categorized GC into four subtypes based on gene expression data, each associated with a distinct clinical outcome: microsatellite unstable, microsatellite stable and epithelial-to-mesenchymal transition (MSS/EMT), MSS/TP53+ (TP53 active), and MSS/TP53- (TP53 inactive). Such molecular profiling data have facilitated the identification of candidate driver alterations in GC, such as gene mutations, chromosomal alterations, and transcriptional and epigenetic changes. However, these nucleic-acid-based analyses provide merely a snapshot of the genomic landscape of cancer cells and fall short of capturing the stromal heterogeneity. This limitation is not negligible, as the dynamically evolving tumor stroma often influences the efficacy of anticancer therapies (4). Hence, recent research underscores the significance of delineating stromal characteristics for precise patient categorization (5, 6, 7).

Extracellular matrix (ECM) proteins within the tumor microenvironment (TME) are pivotal stromal components often overlooked in analyses based on genetic information. Nevertheless, these ECM proteins broadly affect the overall tumor biology, molecular phenotype, and morphology of the tumor, and the sensitivity of antitumor therapy. For example, it has been reported that poorly cohesive carcinomas (PCCs)—specifically, the signet-ring cell phenotype and not-otherwise-specified phenotype (NOS)—are clonally identical but diverge pathohistologically due to the regulatory influence of their distinct stromal feature (8). It highlights the need for in-depth characterization of stromal-centered understanding in GC. To date, there has been a lack of research focused on the ECM-centric perspective and investigations into the specific attributes of tumorous ECM.

To address these issues, we designed an analytical procedure integrating ECM proteomic data with single-cell RNA sequencing (scRNA-seq). Our aims are (i) to distinguish tumorous ECM from that of normal gastric tissue, (ii) to identify ECM proteins associated with malignant phenotypes in GC, and (iii) to refine key cellular player involved in the remodeling of malignant ECM.

## Experimental Procedures

### Experimental Design and Statistical Rationale

A total of 45 tissue samples were obtained from GC tissues, normal adjacent to tumor (NAT) tissues, normal (NN) tissues from noncancerous patients, and lymph node metastasis tissues collected from 23 individuals. After the ECM enrichment process by detergent-based decellularization, quantitative proteomic analysis of decellularized GC tissues was performed by 11-plex tandem mass tag (TMT) spectrometry. TMT-based proteomics data were used for hierarchical clustering, principal component analysis (PCA), differential expressed matrisome protein (DEP), and pairing analysis. The normalized intensity values were scaled and clustered with the matrisome protein data based on the Euclidean distance in Perseus software (www.maxquant.org/perseus) (9). For PCA, only normalized intensity values of matrisome proteins were used. DEPs between the two conditions (tumor *versus* NAT, PCC-NOS *versus* non-PCC-NOS) were determined using Welch’s *t* test. DEPs with fold change (FC) > √2 and *p* < 0.05 were selected. PCC-NOS enriched matrisome proteins (PEMs) were identified as the DEPs between PCC-NOS tumor sample *versus* non-PCC-NOS tumor sample.

The results of ECM proteomics were integrated with single-cell RNA sequencing analysis to reveal the cancer-associated fibroblasts (CAFs) providing ECM heterogeneity. The cellular origins of DEPs were identified using the average expression levels of cell types. Cell type-specific genes were defined using the FindAllMarkers function in the Seurat package; an adjusted *p* < 0.01 was used as a threshold to determine whether the gene expression was cell type-specific. PCC-NOS-specific CAFs were identified by calculating score of PEM expression for each single cell of public scRNA-seq data using sc gene set variation analysis (GSVA) R package. PCC-NOS-specific CAFs were validated by analyzing gene expression of isolated primary CAFs and immunohistochemistry (IHC) of tumor microarray from 114 consecutive GC cases.

### Patient and Tissue Sample Collection

Postoperative specimens from a total of 23 patients who underwent gastric surgical resection between January and August 2021 were employed in this study. Nineteen were GC patients and four were noncancer patients who received sleeve gastrectomy for morbid obesity. Fresh tissue specimens, each measuring less than 2 x 2 cm, were obtained intraoperatively from both tumor and normal tissues contained in the resected specimens by the surgeon. These were then delivered to the laboratory in Dulbecco’s modified Eagle’s medium (GIBCO Life Technologies) containing 5% penicillin/streptomycin and amphotericin B, and were kept on ice. The specimens were washed with PBS also containing 5% penicillin and streptomycin, cut into appropriate pieces, and subsequently stored in cryovials at the human derivatives repository at −195 °C, which was a process approved by the Institutional Review Board of Seoul National University Hospital (IRB No. 2006–052–1131). In total, 45 tissues were obtained: 19 tumor tissues and 17 normal tissues from gastric cancer patients, five lymph node tissues from gastric cancer patients, and four normal tissues from normal (morbid obesity) patients.

This study was conducted in accordance with the provisions of the Declaration of Helsinki for the participation of human subjects in research and the study protocol was approved by the Institutional Review Board of Seoul National University Hospital (IRB No. 2304-144-1427).

### Tissue Decellularization Process

Collected tissues were decellularized using a detergent-based method. The following decellularizing detergent solution was used to remove the cellular components from tissues: 1% (v/v) Triton X-100 (T8787; Sigma-Aldrich) and 0.1% (v/v) ammonium hydroxide (221228; Sigma-Aldrich) in distilled water. Tissue samples were cut into small sections (3 × 3 × 3 mm) and treated with a decellularizing solution for >3 h; the solution was replaced at 30-min intervals or when it became opaque. When the tissue became colorless, the resulting patient-derived tissue ECM (pdECM) samples were washed with Dulbecco’s phosphate buffered-saline (Welgene) for 2 days; the solution was replaced at 1-h intervals. Then, the tissue was washed with distilled water, 4 times for 10 min each, to remove residual Dulbecco’s phosphate-buffered saline. Decellularization was performed on an orbital shaker at room temperature, using a speed of 70 rpm. Finally, pdECM samples were lyophilized for 1 day and stored at −20 °C until use.

### pdECM Characterization

For H&E staining, native tissues and decellularized tissues were fixed in 4% paraformaldehyde (Biosesang) for 1 day and embedded in Paraplast (Leica Biosystems); each sample was cut into 10-μm-thick sections. The sectioned samples were stained with hematoxylin and eosin using the standard protocol with slight modification. The DNA content in pdECM samples was quantified using the DNA extraction kit (Bioneer) by the manufacturer’s recommendations, and DNA concentrations were measured using a DS-11 Spectrophotometer (DeNovix).

### S-Trap Protein Digestion

The S-Trap mini (ProtiFi) was used to perform protein digestion, in accordance with a slightly modified version of the manufacturer’s instructions. Briefly, nearly 5 mg of decellularized gastric tissues were mixed with 1× SDS buffer (5% SDS, 50 mM triethylammonium bicarbonate (TEAB), pH 8.5) and sonicated by VCX 130 (Sonics), as directed by the manufacturer. Each sonicated sample was centrifuged at 13,000g for 10 min. Each supernatant was collected in a 1.5-mL tube and boiled with 20 mM DTT (final concentration) at 95 °C for 10 min. Then, the solution was cooled to room temperature and alkylated with 40 mM iodoacetamide in the dark for 30 min. Subsequently, the sodium dodecyl sulfate lysate was added to 12% aqueous phosphoric acid (1:10 dilution, yielding a final concentration of 1.2% phosphoric acid) and seven volumes of binding buffer (90% aqueous methanol with a final concentration of 100 mM TEAB; pH 7.1). After gentle mixing, the protein solution was loaded onto the S-Trap filter, spun at 3000 g for 1 min, collected using flow-through, and reloaded onto a filter. This step was repeated two times, and the filter was washed three times with 400 μl of binding buffer. Finally, 10 μg of trypsin (Promega) and 125 μl of digestion buffer (50 mM TEAB) were added to the filter at 1:25 w/w and digested at 37 °C for 16 h. To elute the digested peptides, three step-wise buffers were applied, with 80 μl of each peptide repeated once; these buffers included 50 mM TEAB, 0.2% formic acid in water, and 50% acetonitrile/0.2% formic acid in water. The peptide solution was pooled, lyophilized, and desalted in accordance with the protocol of the Pierce Peptide Desalting Spin Column (Thermo Fisher Scientific).

### TMT 11-Plex Labeling

To compare data between samples, multiplexing was used with five sets of TMT11-plexes for 19 tumor tissues and 17 normal tissues from gastric cancer patients, 5 lymph node tissues from gastric cancer patients, and 4 normal tissues from normal (morbid obesity) patients. A pooled common control was constructed as a reference to facilitate combinations of data for multiple sets of TMT11-plexes. The control consisted of equal weights of total peptides from each of the samples used in the experiment. Each TMT set included two reference samples of the same amount labeled with 131N and 131C tags, respectively, for quality checks in TMT experiments, as well as nine tissue samples. In total, 100 μg of desalted peptides were measured using the Pierce Quantitative Fluorometric Peptide Assay kit, in accordance with the manufacturer’s instructions (Thermo Fisher Scientific). The desalted and dried peptides were redissolved in 100 mM TEAB (100 μl) with TMT 11-plex reagents, in accordance with the manufacturer’s instructions (Thermo Fisher Scientific). Next, 0.8 mg of TMT reagent (41 μl) was added to each sample, mixed, and incubated at room temperature for 1 h. The reactions were quenched using 8 μl of 5% hydroxylamine (Thermo Fisher Scientific) and incubated at room temperature for 15 min. The labeled samples (25–100 μg) were combined, dried, and desalted using Pierce Peptide Desalting Spin Columns (Thermo Fisher Scientific). The eluates were dried and stored at −80 °C.

### High pH Reversed-Phase Fractionation

The TMT-labeled peptides were fractionated using a Shimadzu HPLC system that consisted of a binary pump, an autosampler, a degasser, a variable wave detector, and a fraction collector. High pH reversed-phase fractionation was performed using a 4.6 × 150 mm Waters XBridge BEH C18 column (diameter, 2.5 μm). Mobile phase A consisted of 5 mM ammonium formate in 100% water, whereas mobile phase B consisted of 5 mM ammonium formate in 95% acetonitrile. Sample separation was accomplished using the following linear gradient: 5% B for 15 min, from 5% to 15% B over 5 min, from 15% to 40% B over 30 min, 40% B for 5 min, from 40% to 95% B over 4 min, 95% B for 4 min, from 95% to 5% B over 1 min, and 5% B for an additional 9 min. Time-dependent fractions were collected from 21 to 61 min for a total of 40 fractions, yielding approximately 1 ml/fraction. The variable wave detector was monitored at 214 nm. After collection, 40 fractions were combined into 20 fractions by blending fractions (*e.g.*, 1 and 21; 2 and 22; 3 and 23). Each fraction was dissolved in 200 μl water/formic acid (99.9:0.1, v:v) for liquid chromatography with tandem mass spectrometry analysis.

### Nano LC-Electrospray Ionization-MS/MS Analysis

A nano-flow ultrahigh-performance liquid chromatography system (UltiMate 3000 RSLCnano System; Thermo Fisher Scientific) coupled to the Orbitrap Eclipse Tribrid mass spectrometer (Thermo Fisher Scientific) was used for proteome analyses. Fractionated peptides were injected and separated on EASY-Spray PepMap RSLC C18 Column ES803A (2 μm, 100 Å, 75 μm × 50 cm; Thermo Fisher Scientific) operated at 45 °C. A gradient from 5% to 95% mobile phase B was applied over 140 min with a flow rate of 250 nl/min, using mobile phases A (water/formic acid, 99.9:0.1, v:v) and B (acetonitrile/formic acid, 99.9:0.1, v:v). The electrospray ionization voltage was 1800 to 1900 V, and the ion transfer tube temperature was 275 °C.

Ultrahigh-performance liquid chromatography -MS/MS data were acquired using a data-dependent top-speed mode comprising a full scan to maximize the number of MS2 scans during the 3 s of cycle time. The full scan (MS1) was detected using the Orbitrap analyzer at a resolution of 120 K, with a mass range of 400 to 2000 m/z. The automatic gain control target mode was “standard,” the maximum injection time mode was “auto,” the charge states were set at 2 to 6, and a dynamic exclusion window was set at 30 s. The second scan (MS2) was collided by the higher-energy C-trap dissociation mode. The higher-energy C-trap dissociation spectra were detected using the Orbitrap analyzer at a resolution of 30 K with 37% fixed collision energy for isobaric labeled peptides. The maximum injection time mode was “auto,” the isolation window was 0.7, the automatic gain control target mode was “standard,” the first mass was fixed at 110, and the mode was Turbo TMT.

### Data Processing

For proteomics analysis, raw files were converted to MS (.ms1) and MS2 (.ms2) files using RawConverter (https://github.com/proteomicsyates/RawConverter) (The Scripps Research Institute, La Jolla, CA, USA). Proteome search and database generation were conducted using IP2 (Integrated Platform for mass spectrometry data analysis, Bruker). Proteome results were analyzed using ProLuCID, DTASelect2, and Census (https://github.com/lazear/census). The database for analysis was generated using the UniProt human proteome database (20,645 entries, updated on January 01, 2020). The following IP2 parameters were used: precursor 10 ppm and fragment mass tolerance 200 ppm; enzyme, trypsin; miscleavages, ≤2; static modifications, 57.0215 Da added at cysteine, 229.1629 Da added at lysine and N-terminal; differential modifications, 15.9949 Da added at methionine; and minimum number of peptides per protein, 2. Pooled spectral files from all 20 fractions were compared with both normal and reversed databases using the same parameters. For peptide validation, the false positive rate was set to below 0.01 of the spectrum levels. TMT reporter ion analysis was conducted using Census software (https://github.com/lazear/census), with a TMT reporter ion mass tolerance of 20 ppm.

### Normalization of Protein Abundance

Because of differences in sample handling and laboratory environments, there were systematic and sample-specific biases in the quantification of protein abundance. To eliminate these effects, we calculated the median of log2-transformed peptide abundance; column values were subtracted from median values to achieve a common median of 0. Then, we calculated the average of the median values, readded them to the zero-centered column, and transformed the recentered value using the y = 2 ∧ (x) function. For intersample intensity normalization, the relative intensity value of each protein was calculated through division of the intensity values of the proteins in each sample by the original intensity value of the second internal reference (R2) column, which was used as the reference for other samples. Then, the final normalized intensity values were calculated through multiplication of the relative intensity value of each protein by the average normalized intensity value of the R2 column. The normalized value was transformed using the y = 2 ∧ (x) function. The abundance values were used for further proteome analysis.

### Immunohistochemistry

Immunostaining for the following four markers was performed on whole sections of representative gastric cancer cases as previously described (10, 11); serpin family H member 1 (SERPINH1) (=HSP47, Rabbit monoclonal, ab109117, Abcam, 1:300), and hyaluronan and proteoglycan link protein 1 (HAPLN1) (Goat polyclonal, AF2608, Bio-Techne, 1:2000).

ABCA8 IHC was performed on a tissue microarray consisting of 114 consecutive GC cases (Rabbit polyclonal, HPA044914, Sigma-Aldrich, 1:200). The staining intensity of the stroma around the cancer was classified as 0 (no staining), 1 (weak staining), 2 (moderate staining), or 3 (strong staining). The ABCA8 expression level was then classified as ABCA8- (intensity 0 and 1) or ABCA+ (intensity 2 and 3).

### Bioinformatic Data Analysis

scRNA-Seq analysis of GC tissues was performed using published data from a previous study (12). Briefly, single-cell GC dissociates of Singapore cohorts were collected, and the manufacturer’s instructions generated a barcoded sequencing library. Only patients with data classified as intestinal or diffuse according to the Lauren classification were included in the analysis. Specific parameters, reagent kits, and pipelines for sequencing were used as previously described. The previously described markers annotated five major cell types (epithelial, stromal, T, macrophage, and B cells). Within the stromal cells, fibroblasts and endothelial cells were separated by the expression of plasmalemma vesicle associated protein, which was previously suggested.

Next, we identified the cellular origins of DEPs using the average expression levels of cell types. Cell type-specific genes were defined using the FindAllMarkers function in the Seurat (https://satijalab.org/seurat/) package; an adjusted *p* < 0.01 was used as a threshold to determine whether the gene expression was cell type-specific. The cell type-specific average expression levels were determined using the AverageExpression function in the Seurat package; the cell type with the highest average expression level was regarded as the cellular origin of the gene.

We used only fibroblast populations previously annotated with the fibroblast cell type to calculate the PEM score. The gene expression patterns of fibroblasts were normalized and clustered by (1) performing linear dimensional reduction using the RunPCA function in the Seurat package with all matrisome genes regarded as features, (2) using the FindNeighbors function in the Seurat package with the parameter dims = 1:20, (3) using the FindClusters function in the Seurat package with the parameter resolution = 0.5, and (4) using the RunUMAP function in the Seurat package with the parameter dims = 1:20 to plot fibroblasts in the dimensional space. The single-cell level GSVA (scGSVA) R package (https://github.com/guokai8/scGSVA) with the PEM gene list calculated the PEM score of each cell. The Pearson’s correlation coefficient values of each gene were calculated with the PEM score and transcripts per million (TPM) values of each single cell.

The expression of PEM markers on the CAF atlas was identified with the publicly accessible interactive scRNA-seq data provided by Luo *et al*. (13). Cell types were previously annotated, and adipogenic CAF (cluster 4) markers were sorted based on the log2_fold_changes value. The top 30 adipogenic CAF markers were selected for calculating the CAF_adi_ score of samples in TCGA-stomach adenocarcinoma (STAD) bulk RNAseq data.

TCGA-STAD gene expression datasets and a clinical dataset from the TCGAbiolinks (https://bioconductor.org/packages/release/bioc/html/TCGAbiolinks.html) package were collected (14). After the gene expression information had been downloaded from the Illumina platform, the raw counts were converted to normalized TPM values. In total, 375 tumor samples were analyzed. The expression patterns of specific gene sets in each TCGA sample were evaluated using single sample gene set enrichment analysis (ssGSEA) (15). The ssGSEA scores for PEM and CAF_adi_ were calculated using the ssGSEAprojection (https://www.genepattern.org/modules/docs/ssGSEAProjection/4#gsc.tab=0) package in the GenePattern web-based tool. The overall survival (OS) for each marker gene set and *ABCA8* gene were visualized using the KM plotter database (16).

### Primary Fibroblast Isolation and Transcriptomic Analysis

Gastric mucosal tissue samples, acquired in the “Patient and tissue sample collection” section, were processed as described previously (17). Obtained tissue samples were washed with PBS and 5% penicillin and streptomycin, then dissected into 2 to 3 mm squares, washed with PBS three times, and placed at regular distance in 6-well plates. The samples were covered with 22 mm cover glasses (#HSU-0101060) and cultured in Dulbecco’s modified Eagle’s medium (GIBCO Life Technologies) supplemented with 10% fetal bovine serum, 1% penicillin, and streptomycin. Fibroblasts were isolated from gastric cancer tissues (CAFs using an outgrowth method. Isolated fibroblasts were validated by immunochemical staining for alpha smooth muscle actin and fibroblast activation protein, which are representative markers of fibroblasts. All experiments were conducted with fibroblasts under nine passages. RNA was extracted using an RNA prep kit (PureLink RNA Mini Kit; Thermo Fisher Scientific), and complementary DNA was synthesized using a PrimeScript 1st Strand complementary DNA Synthesis Kit (Takara Bio Inc). RNA sequencing was carried out on an Illumina HiSeq2500 sequencer by Theragen Etex Bio Institute. The raw Illumina sequence data were demultiplexed and converted to fastq files. The mRNA sequencing reads were mapped to *Homo sapiens* genome assembly GRCh38.p13 from the Genome Reference Consortium by STAR (https://github.com/alexdobin/STAR) (version 2.7.10). Mapped reads were assembled with known genes and quantified in terms of read counts and sample normalized values, such as fragments per kilobase of transcript per million mapped reads and TPM mapped reads, using RSEM (https://github.com/deweylab/RSEM) (version 1.3.3).

### Survival Analysis

The patients were followed up from the date of surgery to the date of death or the last follow-up for OS. OS was calculated using the log-rank test with Kaplan–Meier curve. All statistical tests were two-sided, and statistical significance was defined as *p* < 0.05.

## Results

### Quantitative Proteomic Analysis of Patient-Derived Tissue ECM (pdECM)

**Fig. 1:**
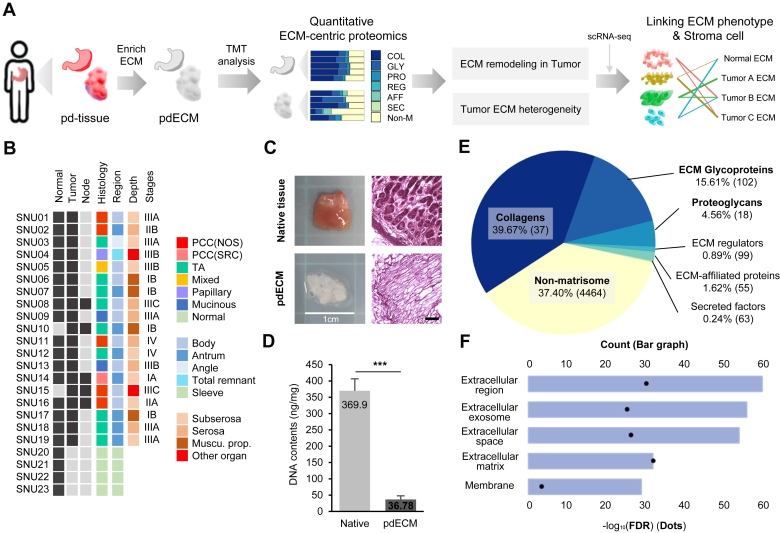
**Characterization of patient-derived decellularized ECM.***A*, scheme of the workflow. Proteomic profiles of patient-derived tissue ECM (pdECM) were identified by tandem mass tag (TMT) mass spectrometry. *B*, clinical data of patients and samples. A histology, a stage of tumor, an anatomical region, and a depth of tumor are shown. *C*, representative image of native tissue and pdECM. The scale bar represents 100 μm. *D*, DNA quantification of native tissue and pdECM. *E*, average intensity of detected proteins in equally mixed reference samples. The counts of proteins within each category are shown in each parenthesis. *F*, the cellular component term of gene ontology of 100 proteins which have the highest intensities were analyzed. The bar graph shows the counts of proteins and the dots shows the statistical significance of each category. ECM, extracellular matrix; pdECM, patient-derived tissue ECM.

To comprehensively analyze the ECM proteomic profiles of normal gastric tissues and histology-annotated GC tissues, we employed decellularization technology for ECM enrichment and quantitative proteomics using TMT technology. In addition to the acellular components, we integrated the transcriptomic analysis of cellular components in the stroma. We adopted a multimodal analysis approach that combined scRNA-seq analysis with ECM proteomics to do this. (Fig. 1*A*)

A total of 45 tissue samples were obtained from GC tissues, NAT tissues, normal (NN) tissues from noncancerous patients, and lymph node metastasis tissues collected from 23 individuals. We observed that there was no bias toward a single type in terms of stages, anatomical region, depth, and histology, indicating the composition of a diverse patient group (Fig. 1*B*). The efficacy of the decellularization process in removing cellular components was confirmed. H&E staining (Fig. 1*C*) and DNA quantification (Fig. 1*D*) demonstrated that the tissue nuclei were effectively removed in pdECM, and a significant reduction in DNA content corresponding to the nucleus was observed.

Using TMT-based quantitative proteomic analysis, we detected a total of 4838 proteins. Based on the matrisome database, 376 proteins correspond to matrisome proteins, including 37 collagens (COLs), 102 ECM glycoproteins (GLY), 18 proteoglycans (PRO), 99 ECM regulators (REG), 55 ECM-affiliated proteins (AFF), and 63 secreted factors (SEC). Analyzing the representative pdECM sample made by a mixture of an equal amount of all samples, 376 matrisome proteins accounted for 62.6% of total intensity, contrasting with 4464 nonmicrosome proteins for the other less proportion (Fig. 1*E*). The top 100 proteins with the highest intensities were investigated by annotating genes with the gene ontology: cellular component (GO: CC) categories using DAVID (https://david.ncifcrf.gov/). Notably, the category that showed the highest significance was identified as being associated with the extracellular region and ECM, indicating a strong enrichment of ECM-related proteins (Fig. 1*F*). These observations suggest that ECM enrichment process enables large-scale quantitative profiling of tissue ECM components.

### ECM Profile of pdECM Shows the Difference Between the Normal Tissues and the Tumor Tissues and Tumor Heterogeneity

**Fig. 2:**
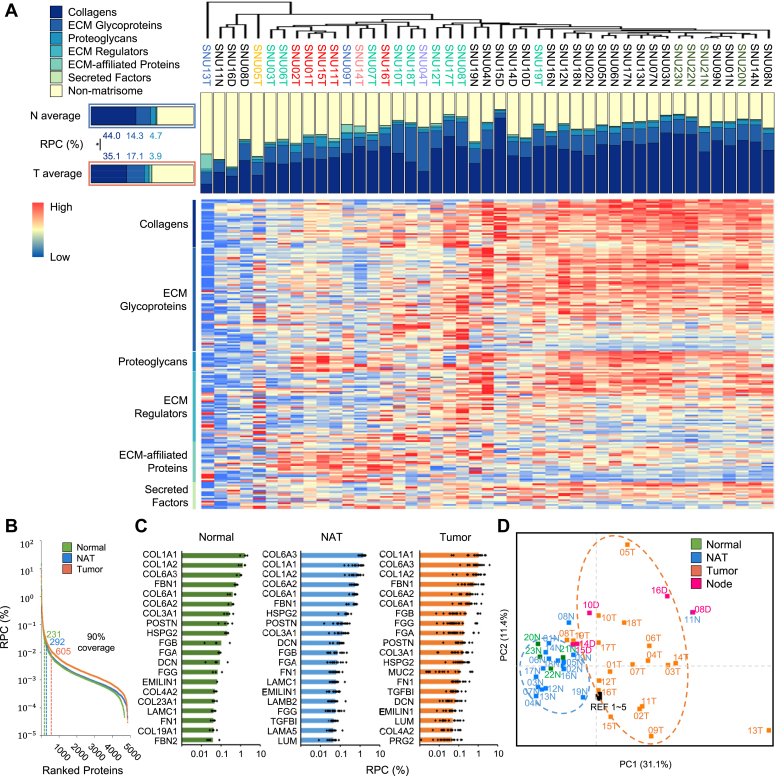
**Matrisome-focused proteomic profiles of patient-derived normal and tumor ECM.***A*, matrisome composition of each pdECM indicated by hierarchical clustered heatmap and bar plot. ∗*p* < 0.05. *B*, composition of proteins ranked by relative percent composition (RPC). The counts of proteins that cover 90% of total intensities are shown by each group. *C*, most abundant 20 matrisome proteins in each group. Bar plot shows the average RPC of each protein in each tissue type. RPC of each protein of each sample are shown as *dots* in the bar plot. *D*, PCA plot with matrisome protein intensities of all samples. PCA, principal component analysis; pdECM, patient-derived tissue ECM.

Hierarchical clustering using the intensities of matrisome proteins revealed distinct clusters separating the normal tissues (NN and NAT tissues) from the tumor tissues, indicating differential ECM profiles between normal and tumor tissues (Fig. 2*A*). The relative percentage composition (RPC) of the main categories of matrisome was compared between normal and tumor tissues. A significant increase in COL content was observed in the normal tissues, as well as a higher PRO content. On the other hand, GLY showed higher levels in the tumor tissues (Fig. 2*A*).

The average RPC of all detected protein by each condition were sorted in descending order (Fig. 2*B*). The number of proteins account for 90% of the cumulative RPC, the NN tissues had 231 proteins, and the NAT tissues had 292 proteins. In contrast, the tumor tissues had 605 proteins, which differed from the normal tissues. Also, the 20 most abundant matrisome proteins were identified as described in Figure 2*C*. In all conditions, the top six matrisome proteins were the same COL1 family, COL6 family, and fibrillin1 (FBN1). However, the composition among the tissues was slightly different. In the tumor tissues, fibrinogen family (FGA/FGB/FGG), mucin 2 (MUC2), and transforming growth factor beta induced (TGFBI) proteins were ranked higher than other tissues. Meanwhile, heparan sulfate proteoglycan 2 (HSPG2), decorin (DCN), and laminin subunit gamma 1 (LAMC1) were ranked higher in the normal tissues.

The dimensional reduction with the ECM profile of samples showed the differences more clearly between the normal and tumor tissues. In the normal tissues, the ECM profiles of the samples showed similarity across both NN and NAT tissues, distinctly differing from those of the tumor tissues, with the exception of one sample. In contract, the ECM profiles of the tumor tissues not only diverged from those of the normal tissues but also exhibited considerable variability among the tumor tissues (Fig. 2*D*). The heterogeneity of the tumor ECM was further highlighted by analyzing the distribution of the top 20 proteins in each sample type (Fig. 2*C*). Tumor tissues exhibited a notable wider distribution of these proteins compared to normal tissues. These results reveal the distinct ECM profile of tumor tissues relative to normal tissues and the presence of variability within the tumor ECM.

### Differentially Expressed Matrisome Proteins Between the NAT and Tumor Tissue

**Fig. 3:**
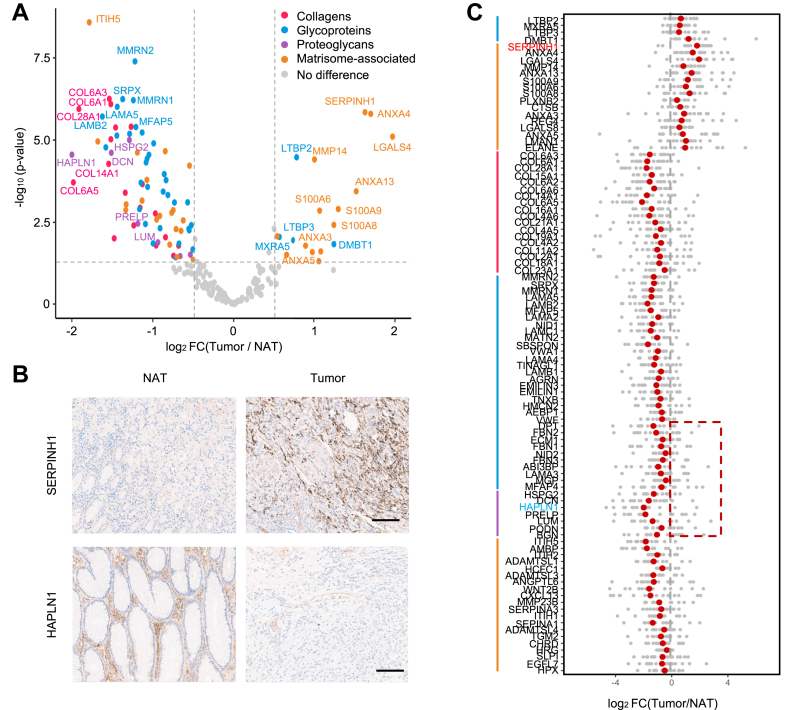
**Differentially expressed matrisome proteins (DEPs) between adjacent normal and tumor ECM.***A*, volcano plot of differentially expressed matrisome proteins between adj. normal and tumor ECM. *Dotted lines* show thresholds for DEPs. (|log_2_FC| > 0.5 and *p*-value <0.05). *B*, representative immunohistochemistry (IHC) image of SERPINH1 and HAPLN1 which are tumor-enriched and normal-enriched matrisome each. *C*, paired analysis of DEPs. *Gray dots* show the log_2_FC of each patient and *red dots* show the median values. Dots in *red box* show the patients with the opposite tendency. ECM, extracellular matrix; FC, fold change; HAPLN1, hyaluronan and proteoglycan link protein 1; SERPINH1, serpin family H member 1.

DEPs were identified between the NAT and the tumor tissues. FC values (average tumor/average NAT intensity) and *p*-values were calculated, and DEPs were defined with the statistical criteria (|log2FC| > 0.5, *p*-value <0.05) (Fig. 3*A*). The FC values and *p*-values of matrisome proteins were recorded in the supplemental Table S5.

There were 20 tumor-enriched DEPs and 77 NAT-enriched DEPs. Tumor-enriched DEPs include four glycoproteins, SERPINH1, annexin family (ANXA3/4/5/13), S100A family (S100A6/8/9), MMP14, and other matrisome-associated proteins. In COL proteins, increased expression was observed for COL6 family, COL14A1, COL15A1, and COL28A1. For GLY proteins, significant upregulation was observed for multimeric 1/2 (MMRN1/2), sushi repeat-containing protein X-linked (SRPX), laminin subunit alpha 5 (LAMA5), laminin subunit beta 2 (LAMB2), microfibril-associated protein 5 (MFAP5), and other proteins. Interestingly, no specific proteoglycans were found to be more highly expressed in the tumor tissue, while in the NAT tissue, heparan sulfate proteoglycan 2 (HSPG2), decorin (DCN), hyaluronan and proteoglycan link protein 1 (HAPLN1), proline- and arginine-rich end leucine-rich repeat protein (PRELP), lumican (LUM), and other proteins showed enriched expression. *In vivo*, SERPINH1 and HAPLN1 were validated to tumor-enriched and NAT-enriched proteins, each with IHC (Fig. 3*B*).

Paired analysis with the DEPs showed the same trend with FC-based analysis. The median values of the FC of DEPs in all patients were concordant with the trend of DEPs (Fig. 3*C*). Notwithstanding, because of tumor heterogeneity, there were some samples which do not follow the trend with some proteins (like a red box in Fig. 3*C*). These findings highlight the importance of further studies on tumor heterogeneity within the ECM, despite valuable insights from identifying DEPs between normal and tumor tissues.

### PCC-NOS Type Tumor has a Distinct ECM Profile That Distinguishes it From Other Histological Types

**Fig. 4:**
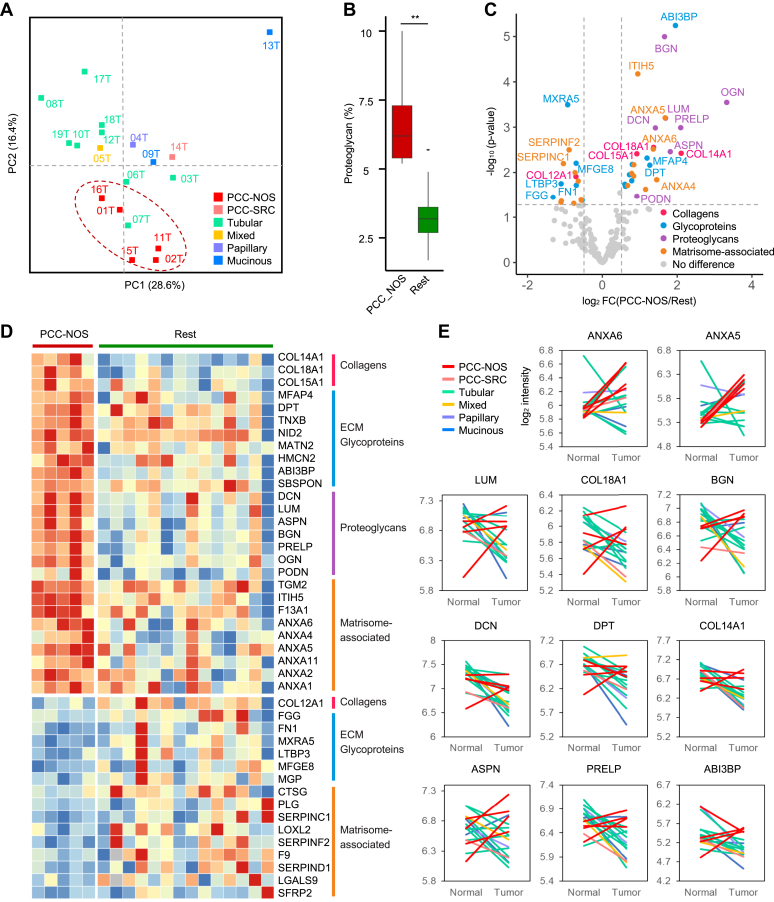
**PCC-NOS specific ECM profile.***A*, PCA plot of tumor ECM with matrisome protein expression profile. *B*, box plot of proteoglycan expression in PCC-NOS type and non-PCC-NOS type. *C*, volcano plot of differentially expressed matrisome proteins (DEPs) between PCC-NOS type and non-PCC-NOS type. *Dotted lines* show thresholds for DEPs. (log_2_FC > 0.5 and *p*-value <0.05). *D*, heatmap of DEPs between PCC-NOS type and non-PCC-NOS type. PCC-NOS enriched matrisome proteins were defined as PEMs. *E*, paired analysis of selected PEMs. The changes of log_2_ (intensity) value are shown by histology. ECM, extracellular matrix; PCA, principal component analysis; PCC-NOS, poorly cohesive carcinoma-not otherwise specified; PEM, PCC-NOS-enriched matrisome protein.

To examine the tumor ECM heterogeneity, the dimensional reduction of ECM profiles with only tumor samples was conducted, and histological information was overlaid (Fig. 4*A*). Interestingly, the samples of PCC-NOS type showed close location that sets it apart from other types.

First of all, with the categories of matrisome, PRO contents of PCC-NOS type were significantly higher than non-PCC-NOS type (Fig. 4*B*). In the case of PCC-NOS type, average PRO content was 6.8%, but with non-PCC-NOS-type was 3.3%. Interestingly, the value of PCC-NOS (6.8%) was much higher than the average PRO content value of the NAT tissues (4.7%) that enriched PRO characterize the PCC-NOS type.

DEPs between PCC-NOS and non-PCC-NOS types were identified based on FC values and *p*-values, as shown in Figure 4*C*. A total of 27 matrisome proteins were enriched in the PCC-NOS type, compared to 16 in the non-PCC-NOS type (Fig. 4*D*). The PCC-NOS type was predominantly enriched with proteoglycans, primarily featuring small leucine repeat proteins (SLRPs). Additionally, various members of the annexin family (ANXA1/2/4/5/6/11), ABI family member 3 binding protein (ABI3BP), microfibril-associated protein 4 (MFAP4), dermatopontin (DPT), and types COL14/15/18A1 COL were significantly upregulated in the PCC-NOS type.

Moreover, the paired analysis involving PEMs revealed distinct cancer-associated ECM characteristics not observed in other subtypes. In most tissues classified as PCC-NOS type, PEM expression was elevated in tumor condition within the same patient. In contrast, in other types, PEM expression noticeably declined (Fig. 4*E*). These findings indicate that ECM features vary across histological subtypes and that PCC-NOS type tissues, in particular, display a unique set of enriched ECM proteins.

### PCC-NOS-specific ECM Proteins are Predominantly Expressed in Adipogenic Cancer-Associated Fibroblasts

**Fig. 5:**
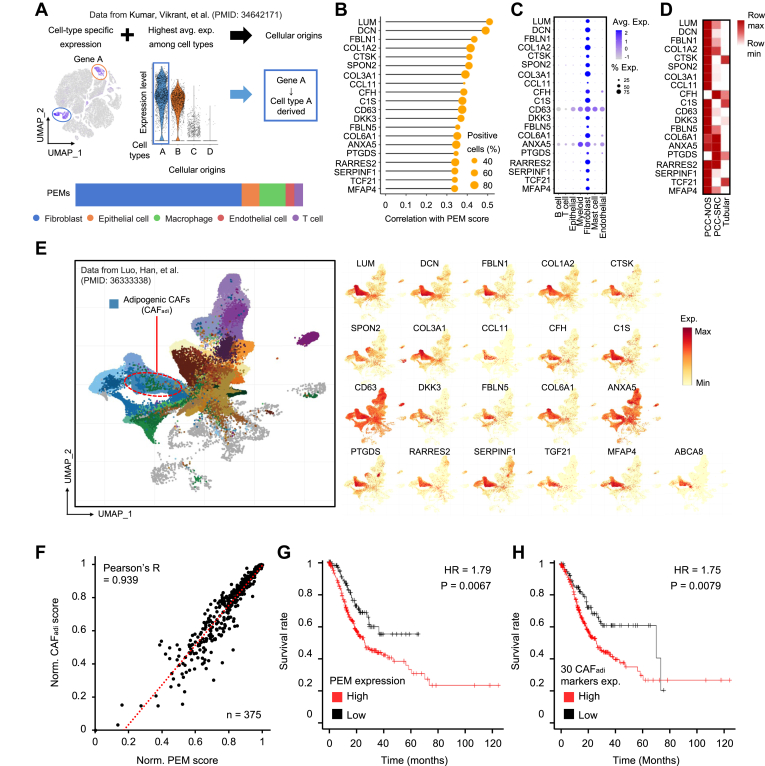
**Identifying PEM mainly expressing cells.***A*, defining cellular origin of proteins with single-cell RNA sequencing. The cellular origin of proteins was identified with two criteria. (1) an expression of gene that encoding the protein is cell type specific. (2) among the cell types, the average expression of the gene is highest in the cellular origin. The cellular origins of PEMs were identified from the data of Kumar *et al*. (12). *B*, lollipop plot of 20 most correlative genes with the PEM score. *Lines* show the Pearson’s correlation coefficient and *circles* show the proportion of positive fibroblasts expressing the genes. *C*, dot plot of single-cell level gene expression of 20 most correlative genes with the PEM score. *D*, the gene expression of 20 most correlative genes in the isolated fibroblasts. *E*, the gene expression of 20 most correlative genes and ABCA8 on pan-cancer single cell analysis from the data of Luo, *et al*. (13) Most of them shows the enriched expression on the adipogenic CAFs (CAF_adi_) region. *F*, scatter plot of the correlation between CAF_adi_ score and PEM score. TCGA STAD data (n = 375) was used for the scoring. *G* and *H*, survival analysis with PEM score and 30 CAF_adi_ markers. The mean expression of selected genes was used to separate the high and low expression of patient group. ABCA8, ATP binding cassette subfamily A member 8; CAF_adi_, adipogenic cancer-associated fibroblast; PEM, PCC-NOS-enriched matrisome protein; STAD, stomach adenocarcinoma; TCGA, the Cancer Genome Atlas.

Fibroblasts, which mainly produce ECM protein, could also affect the unique PCC-NOS ECM profile. Public scRNA-seq data were analyzed to examine the expression of PEMs at the single-cell level (12) (Methods in detail). Single cells were annotated with seven major cell types (fibroblasts, epithelial cells, macrophages, endothelial cells, T cells, B cells, and mast cells), and the cell types mainly expressing PEMs were identified (Fig. 5*A*). Over 70% of PEMs were mainly expressed in fibroblasts.

Based on the expression of genes that encode PEMs, the degree of PEM expression of each fibroblast was calculated as a score by the GSVA (https://bioconductor.org/packages/release/bioc/html/GSVA.html) package. Cells with higher PEM scores were more closely related to the associated fibroblasts. The correlation between gene expression and PEM score was evaluated with Pearson’s correlation coefficient value (Fig. 5*B*). LUM was the most correlated gene with the PEM-associated fibroblast. These highly correlated genes are used as markers of PCC-NOS type. Also, the genes with a low proportion of positive fibroblasts expressing them, such as CCL11 and FBLN5, were used to distinguish PCC-NOS subtypes based on their expression status.

To confirm the fibroblast specificity of PEM-correlated genes, the average and percent expression of genes were validated with seven major cell types. Most PEM-correlated genes were expressed in the fibroblast (Fig. 5*C*). To check the PCC-NOS specificity of PEM-correlated genes further, we isolated primary fibroblasts from specimens representing each histological subtype (PCC-NOS/PCC-signet-ring cell phenotype/tubular adenocarcinoma) and carried out bulk RNA sequencing. Notably, the PEM-correlated genes were expressed at much higher levels in the PCC-NOS subtype than in other types, indicating enrichment of PEM-associated fibroblast in the PCC-NOS subtype (Fig. 5*D*).

We analyzed to explore the relationship between the PEM-associated fibroblasts and the well-recognized fibroblast subtypes in tumors. We used the publicly accessible scRNA-seq data provided by Luo *et al*. as reference data (13). Most PEM-associated genes were highly upregulated in a specific cluster classified as an adipogenic CAF (CAF_adi_) (Fig. 5*E*).

We compared molecular similarities between PEM-associated fibroblasts and CAF_adi_. We used bulk RNA sequencing data from TCGA, focusing on the expression of PEMs and the top 30 markers for CAF_adi_ with the highest log2 FC values. PEM score was positively correlated with CAF_adi_ score (Fig. 5*F*). These two molecular panels (PEMs and CAF_adi_ markers) have clinical implications; elevated PEMs and CAF_adi_ markers expression were associated with a poor prognosis in GC patients (Fig. 5, *G* and *H*). Importantly, the correlation with PEMs and CAF_adi_ markers suggests that PEM-associated fibroblasts may represent a subset of CAF_adi_, and their elevated expression correlates with a poorer prognosis in gastric cancer patients, highlighting the clinical relevance of these findings.

### ABCA8-Positive Fibroblasts, Specific to the PCC-NOS Subtype, Are Associated with Poor Prognosis

**Fig. 6:**
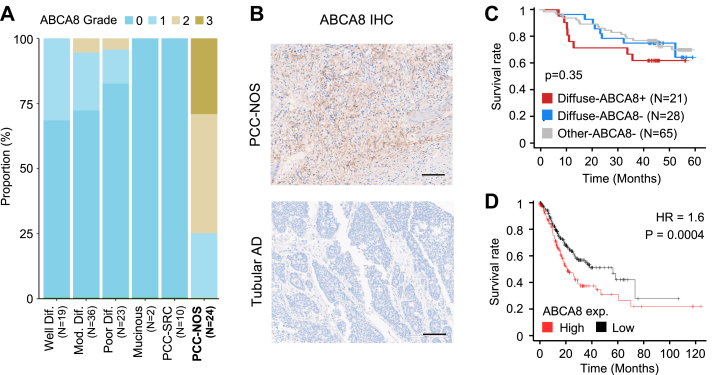
**ABCA8 positive fibroblasts are specific to PCC-NOS subtype and predict poor prognosis.***A*, the bar plot shows the correlation of the histological subtype and ABCA8 grade in tumor microarray IHC analysis. *B*, the representative image of ABCA8 positive fibroblasts in PCC-NOS subtype and ABCA8 negative tissue in tubular adenocarcinoma. The scale bar represents 100 μm. *C*, survival analysis with three groups of patients based on stromal expression of ABCA8 protein. Diffuse-ABCA8+ groups showed poorer survival rate compared with the other diffuse type group and the other histology type group. *D*, survival analysis with *ABCA8* gene expression. *ABCA8* high expressing group showed poorer prognosis. PCC-NOS, poorly cohesive carcinoma-not otherwise specified; IHC, immunohistochemistry; ABCA8, ATP binding cassette subfamily A member 8.

To detect the CAF_adi_ cells selectively in the tumor section, we investigated only the cell-surface marker of CAF_adi_ but not the matrisome markers. In the top 30 markers for CAF_adi_ with the highest log2 FC values, we selected ATP binding cassette subfamily A member 8 (ABCA8) protein for CAF_adi_ marker protein because the expression of *ABCA8* gene is specific to fibroblast but not to the other cell type. With IHC analysis of the tumor microarray, we identified the grade of ABCA8 protein expression with the tumor tissue of 114 GC patients. Surprisingly, only the group of PCC-NOS subtype dominantly expressed the highest grade of expression and most of the patients in the other histological groups showed no expression of ABCA8 protein (Fig. 6*A*). Furthermore, ABCA8 expression was primarily noted in the stromal region, not in cancer cells, reinforcing the specificity of ABCA8-positive fibroblasts to the PCC-NOS subtype. (Fig. 6*B* and Supplemental Fig. S1).

The 114 GC patients were classified using Lauren's classification (diffuse/nondiffuse others) and subsequently, survival analysis was performed based on the expression level of stromal ABCA8 (Fig. 6*C*). The diffuse-ABCA8+ group, while not statistically significant, exhibited a marked decrease in survival around 10 months post follow-up, diverging from the survival curves of both diffuse-ABCA8-and other-ABCA8-groups. Furthermore, patients with high expressing *ABCA8* gene showed poorer prognosis (Fig. 6*D*). The trend toward an association of ABCA8 with poor outcome demonstrates the potential for ABCA8 expression to be a marker of GC with a poor prognosis. (Supplemental Table S1)

## Discussion

The advent of molecular diagnostics has opened new avenues for devising precision treatment strategies and prognostic tools in GC (18). Most of these diagnostic approaches are predicated upon understanding mutations intrinsic to cancer cells or discerning expression patterns of oncogenic genes (1, 19). However, with the new paradigms such as immunotherapy and interventions targeting the TME, there is an elevating demand for precision diagnostics that account for the unique molecular complexities associated with the TME.

Traditional stratification methodologies founded on the histology of the cancer tissues have offered a holistic perspective of the TME. Specifically, a high stromal reaction, as observed histologically, shows a significant correlation with patient treatment outcomes and prognosis in GC (20, 21). Among the TMEs, the ECM serves as a key determinant of stromal features and profoundly impacts cancer cell malignancy, thereby serving as a key cancer hallmark. In present study, we identified prominent up-related and down-related ECM markers as SERPINH1 and HAPLN1, respectively. SERPINH1 is abnormally expressed and has been suggested as a potential prognostic biomarker in stomach adenocarcinoma (22). SERPINH1 is responsible for producing the HSP47 protein, a critical component for ensuring proper COL folding and secretion (23). Unlike the prevailing research has examined the molecular function of SERPINH1 within epithelial cancer cells, our study provides compelling evidence that SERPINH1—a frequently overexpressed ECM protein in tumor tissues—may be localized to stromal cell (*i.e.*, fibroblasts) rather than cancer cells. In addition to the tumor-up expressed protein, we identified tumor-down expressed proteins such as HAPLN1. HAPLN1 is an ECM crosslinker between a hyaluronic acid and chondroitin sulfate proteoglycan. Despite recent findings that CAF-derived HAPLN1 promotes tumor invasion in gastric cancer (24), our data reveal that HAPLN1 is predominantly expressed in NAT tissues, with expression levels sharply decreasing in tumor tissues. scRNA-seq further corroborated that HAPLN1-expressing cells are largely fibroblasts of normal origin; a phenotype conspicuously absent in cancer tissues. Given these findings, future investigations into the potential tumor-suppressive functions of normal-fibroblast-derived HAPLN1 are both warranted and crucial.

Our stroma-focused analysis suggests reevaluating previous research that did not account for the cellular sources of cancer-associated proteins. As gene expression profiling is a reliable, unbiased technology (25, 26), identifying molecular features originating from the stroma that correspond to traditional histological subtypes could offer a new approach for patient stratification. We unveiled a substantial difference in the ECM profile among the histologic subtypes. LUM, COL18A1, BGN, PRELP, and ASPN showed increased expression in tumor tissue compared to the paired NAT tissue, inconsistent with the declined trend in other histologic subtypes. The stroma heterogeneity had been neglected, to identify tumor-associated markers and only the average expression was compared to NAT tissue expression. Our ECM panel for PCC-NOS includes SLRPs like LUM, BGN, PRELP, and ASPN, which exhibit dual functions in cancer. While the SLRPs are known to inhibit tumor growth by blocking growth factor signals like transforming growth factor-beta, their interaction with integrins promote tumor progression through invasion, epithelial-mesenchymal transition, and metastasis (27). Thus, the impact of these proteins on tumor behavior varies with the histological and genotypic/phenotypic traits of cancer.

Recent research has highlighted the significant role of CAFs in GC pathophysiology, particularly in promoting cell proliferation, migration, and metastasis (28). Emerging evidence suggests that different CAF subtypes distinctly influence cancer biology and carry unique prognostic values (29, 30), yet their correlation with various patho-histological GC subtypes remains underexplored. Shah *et al*. (31) identified three specific GC subtypes—proximal, diffuse, and distal—characterized by unique gene expression profiles, with a notable distinction in *ABCA8* expression between diffuse and distal nondiffuse gastric cancers Our study further refined this understanding, revealing that ABCA8 expressing fibroblasts were predominantly found in PCC-NOS tissue, a category of diffuse GC. Notably, the presence of the ABCA8-positive CAF subset is correlated with a poor prognosis in GCs.

In summary, we have performed a comprehensive analysis of the ECM in patients with gastric cancer. Using TMT-based proteomics, we have identified specific ECM profiles in both normal and tumor tissues, revealing distinct patterns associated with each. Moreover, the ECM proteomic analysis has enabled us to differentiate between GC subtypes representing histological characteristics. These approaches propose a stroma-focused classification system for gastric cancer, enhancing our comprehension of disease biology and aiding in the identification of unique TME features specific to each gastric cancer subtype.

## Data Availability

The data supporting the findings of this study are available within the paper and its Supplementary Information. All data are available on request from the authors. All MS data and search results files were deposited in the ProteomeXchange Consortium *via* the MassIVE partner repository with the accession code PXD048518 and MSV000093859 for MassIVE.

## Supplemental data

This article contains supplemental data.

## Conflict of interest

The authors declare no competing interests
