# Supplementary material for: Proteomic Heterogeneity of the Extracellular Matrix Identifies Histologic Subtype-Specific Fibroblast in Gastric Cancer
Source: Mol Cell Proteomics. 2024 Sep 19;23(10):100843. doi: 10.1016/j.mcpro.2024.100843 (PMC11526087; doi:10.1016/j.mcpro.2024.100843)
Supplement: Supplementary Data [file mmc1.pdf]

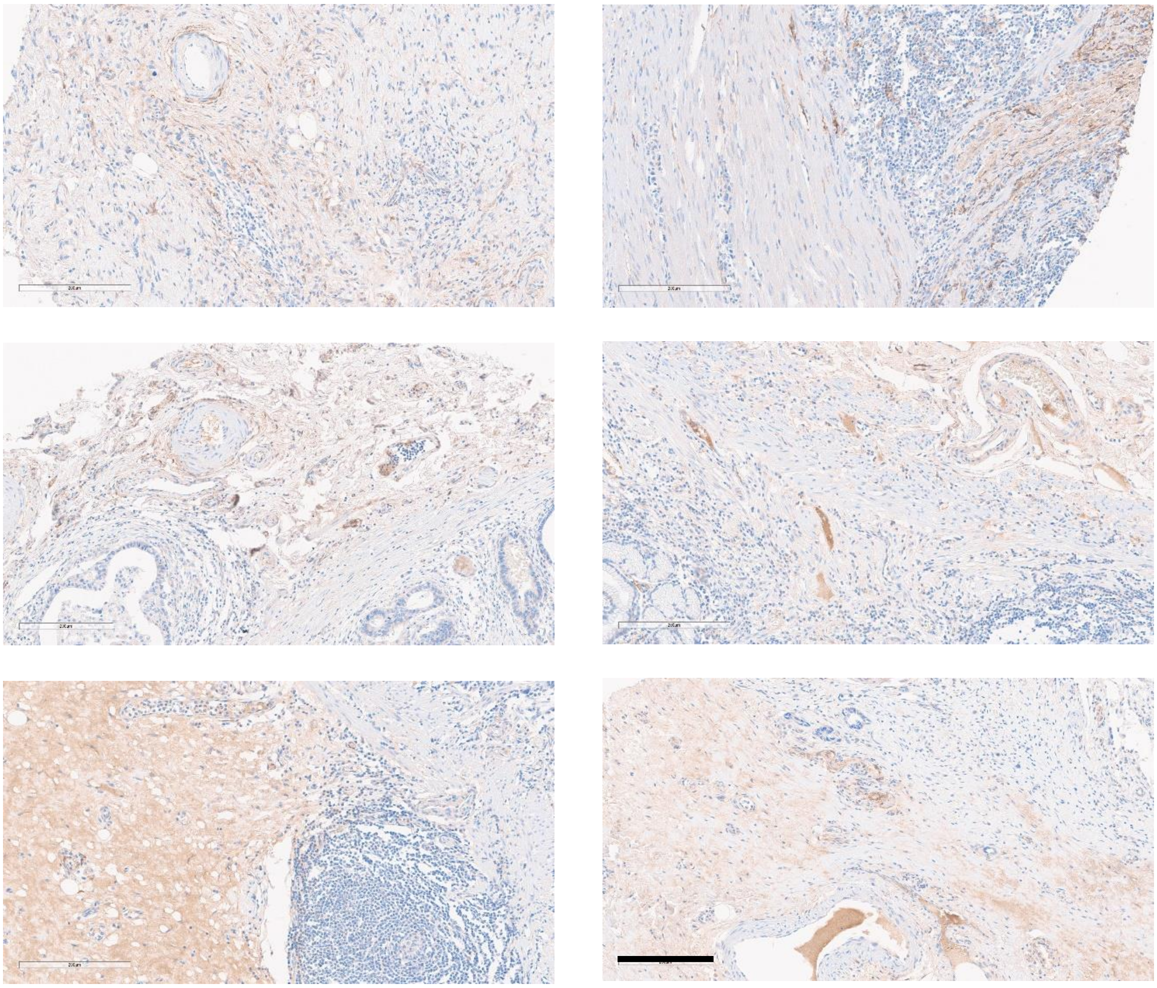

Supplementary Figure 1. Magnified IHC images of ABCA8 expression Scale Bar. 200μm

**Variables**	**ABCA8 negative n (%)**	**ABCA8 positive n (%)**	***P*-value**
**pT stage**			
pT1	35 (92.1)	3 (7.9)	***0.032**
pT2-4	58 (76.3)	18 (23.7)	
**pN stage**			
pN0	53 (91.4)	5 (8.6)	****0.006**
pN1-3	40 (71.4)	16 (28.6)	
**Lymphatic invasion**			
Not identified	53 (84.1)	10 (15.9)	0.295
Present	40 (81.6)	11 (21.6)	
**Venous invasion**			
Not identified	78 (83.9)	15 (16.1)	0.154
Present	15 (71.4)	6 (28.6)	
**Perineural invasion**			
Not identified	66 (91.7)	6 (8.3)	*****<0.001**
Present	27 (64.3)	15 (35.7)	

Supplementary Table 1. ABCA8 protein expression and variables indicative of aggressive disease. Statistical significance with chi-square test, * : <0.05, ** : <0.01, *** : <0.001
